# Incidence of the Triple Whammy Phenomenon among Cardiovascular diseases patients in Saudi Arabia and awareness among healthcare professionals

**DOI:** 10.3389/fneph.2025.1494459

**Published:** 2025-05-27

**Authors:** Mohammad Bonyan Alsobaie, Lubna Alsheikh

**Affiliations:** ^1^ Department of Pharma D, College of Pharmacy, The University of Arizona, Tucson, AZ, United States; ^2^ Pharmaceutical Care Department, The Ministry of Health, Jeddah, Saudi Arabia; ^3^ Department of Biochemistry, Faculty of Science, King Abdulaziz University, Jeddah, Saudi Arabia

**Keywords:** NSAIDs, CVD, triple whammy, angiotensin receptor blockers (ARBII), awareness program, AKI, Diuretics, ACEIs

## Abstract

Cardiovascular diseases are a leading cause of mortality in Saudi Arabia, accounting for approximately 42% of deaths. The “triple whammy” phenomenon—which combines angiotensin-converting enzyme inhibitors or angiotensin receptor blockers, diuretics, and non-steroidal anti-inflammatory drugs—increases the risk of acute kidney injury, particularly in hypertensive patients. This study, which was conducted in small-scale hospitals in Jeddah from 2017 to 2022, assessed the incidence of the triple whammy phenomenon and the awareness of healthcare professionals of this condition. Of 5,654 patient records, 1,899 met the inclusion criteria, with 2.7% experiencing the triple whammy. A survey of 56 healthcare professionals revealed 75% unawareness, with pharmacists and dentists being the most affected. Access to over-the-counter non-steroidal anti-inflammatory drugs and gaps in training likely drive the incidence and awareness deficits. This phenomenon can lead to acute kidney injury, with mortality rates as high as 50%–80% in critically ill patients, and imposes significant costs, representing 5% of hospital budgets and 1% of the overall health expenditure. Interventions including education, pharmacist roles, and non-steroidal anti-inflammatory drug regulation are proposed. Limitations include the small-scale focus and the low survey sample, necessitating national studies to accurately measure incidence and to improve patient safety.

## Introduction

Cardiovascular diseases (CVDs) comprise the leading cause of mortality worldwide. In Saudi Arabia, CVD accounts for approximately 42% of deaths (1). Patients with hypertension and congestive heart failure require medications such as renin–angiotensin system inhibitors [e.g., angiotensin-converting enzyme inhibitors (ACEIs) and angiotensin receptor blockers (ARBs)] and diuretics to manage their conditions effectively while minimizing risks. However, their combination with non-steroidal anti-inflammatory drugs (NSAIDs)—known as the “triple whammy” (TW) phenomenon—has been associated with an increased risk of acute kidney injury (AKI), often leading to hospitalization and increased healthcare costs (2).

TW arises from hemodynamic effects: ACEIs/ARBs dilate efferent arterioles, reducing the glomerular filtration rate (GFR), while NSAIDs constrict afferent arterioles, reducing renal perfusion. Diuretics induce hypovolemia, which exacerbates renal injury (3). TW is particularly common in elderly patients (>75 years), those with liver disease, congestive heart failure, or volume depletion (e.g., vomiting, sepsis, diarrhea, and dehydration) (4). Studies have estimated that TW occurrence can be as high as 30% in critically ill hypertensive patients, with mortality rates reaching 50%–80% in these cases (5). In addition, the costs of AKI from extended hospital stays, closer monitoring, and dialysis represent 5% of hospital budgets and 1% of the overall health expenditure (6). Recent studies have shown that hypertension increases cardiovascular risk, with factors including blood pressure management and obesity metrics linked to adverse outcomes in diverse populations, necessitating careful management in high-risk groups such as those at risk of TW (7, 8).

However, there are no conclusive data on TW incidence in Saudi Arabia. In addition, the awareness of healthcare professionals of TW remains unclear, raising concerns about the unmonitored use of over-the-counter (OTC) NSAIDs. This study, which was conducted in small-scale hospitals in Jeddah, evaluates TW incidence and awareness in order to inform clinical practice and policy.

## Methodology

### Study design and setting

Following ethical approval by the Saudi Arabian Ministry of Health (MOH), this retrospective study was conducted in three MOH-run hospitals in Jeddah, representing a small-scale hospital setting:

1. King Abdullah General Hospital (KAH);
2. King Fahd General Hospital (KFH); and
3. King Abdulaziz Oncology Hospital (KAOH).

This study aimed to evaluate the incidence of TW among hypertensive patients and to assess the awareness of healthcare professionals about this condition.

### Data collection

Data were extracted from the electronic health records (EHRs) of patients with chronic diseases from 2017 to 2022, chosen to reflect recent trends post-TW recognition. The inclusion criteria were as follows:

- Patients prescribed ACEIs/ARBs with diuretics;
- Patients with documented NSAID use in addition to ACEIs/ARBs and diuretics, who were classified into TW cases (group 1);
- Patients prescribed ACEIs/ARBs with diuretics, but no recorded NSAID use, who were classified as a high-risk for TW (group 2); and
- Patients not receiving these combinations, who served as controls (group 3).

The questionnaire, designed using straightforward, direct questions, assessed whether healthcare providers (i.e., physicians, pharmacists, dentists, and nurses) were familiar with TW. It evaluated theoretical understanding and case-based scenario accuracy to differentiate familiarity from practical knowledge. As small-scale hospitals were included, only 56 professionals were surveyed, with 14 aware respondents examined in scenarios.

Records for the following were excluded:

- Chronic kidney disease (CKD) or renal failure;
- Patients <20 years old, due to a lower TW risk; and
- Incomplete or missing medication history.

### Statistical analysis

Data were analyzed using SPSS (version 22) software. Descriptive statistics (frequency, percentage, and the mean ± SD) were used to describe the variables. Comparative analyses utilized the Kruskal–Wallis test for non-normally distributed continuous variables and the chi-square test for categorical data. A *p*-value <0.05 was considered significant.

## Results

**Figure 1:**
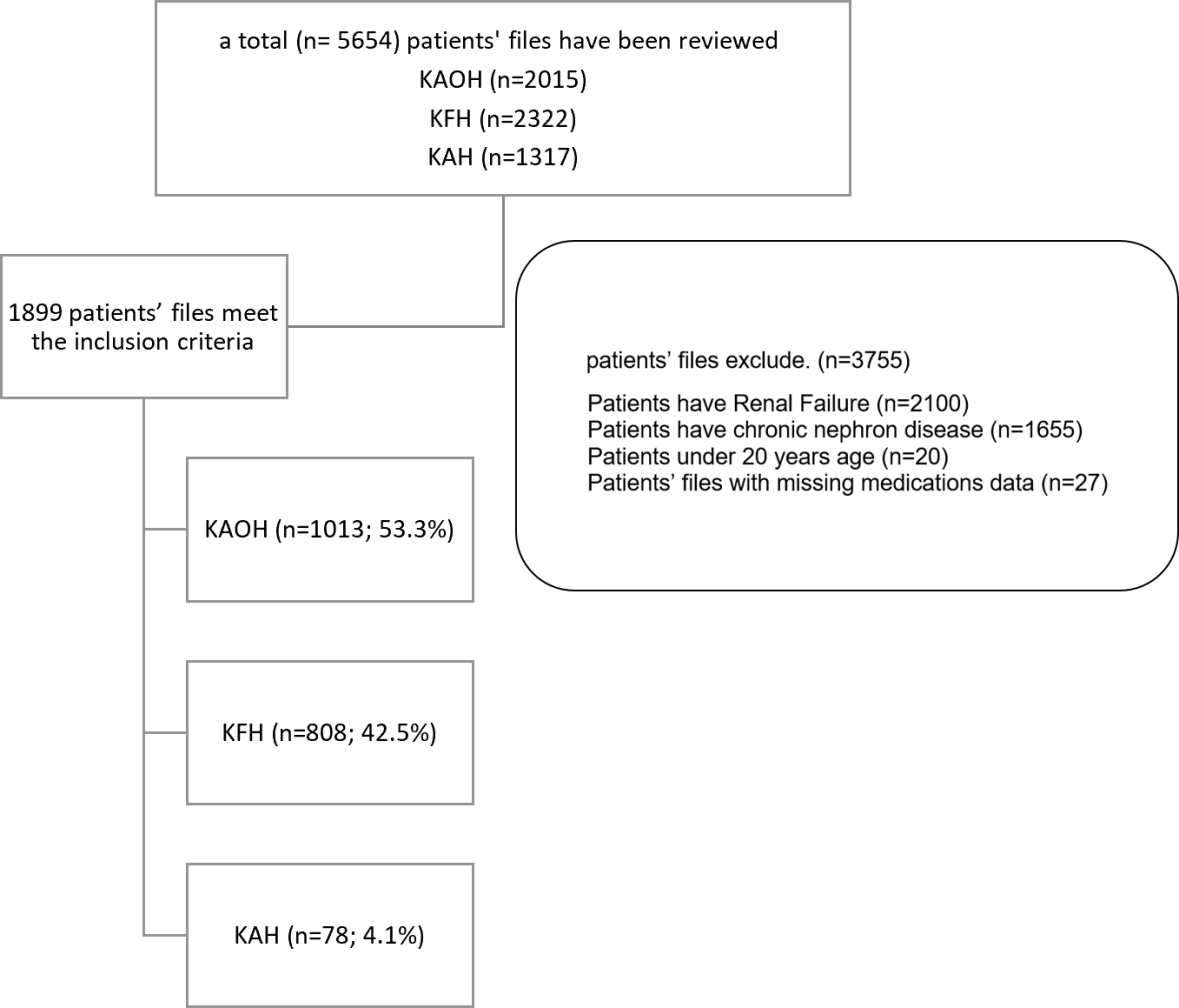
Flow chart of cases inclusion and exclusion from data that collected from hospitals. KAOH: King Abdulaziz Oncology Hospital, KFH: King Fahad General Hospital and KAH: King Abdullah Hospital, 1899 patients' health records met the inclusion criteria 53.3% from KAOH, 42.5% KFH and 4.1% KAH.

A total number of patients' health records who have chronic disease between 2017-2022 were more than five thousand (n= 5654) as following; KAH (n=1317), KFH (n=2322) and KAOH (n=2015). All these electronic records of the patients were reviewed and 3755 of them were excluded for different reasons as prescribed on **Figure 1**.

The incidence of TW phenomena (group1) was notably the highest in KFH with 86.5% , while KAH 11.5% and KAOH 1.9% (P-value <0.001). Also, Patients have a combination may use NSIAD from OTC or different providers lead to TW (group 2) was 73.4% in KFH, for KAH and KAOH were 20.6% and 6.0% respectively. While the average of patients with no combination (group 3) was highest in KAOH with 66.2% and 33.8% in KFH; notably, KAH has no representation in the group (P-Value<0.001) **Table 1**.

**Table 1:** Demographic characteristics of inclusion cases that collected from hospitals.

Variables	Group 1 (n=52)n; %	Group 2 (n=349)N; %	Group 3 (n=1498)n; %	P-value
Age in all hospitals (Mean±SD)	65 ± 13.2	62 ±13.0	59 ±15.9	0.000
KAOH	66 ± 0.00	62 ± 15.5	60 ± 15.6	0.403
KFH	67 ± 13.2	61 ± 13.0	56 ± 16.2	0.000
KAH	55 ± 9.2	64 ± 12.2	–	0.054
Gender				
Male	824; 55.0%	236; 67.6%	27; 51.9%	0.143
Female	674; 45.0%	113; 32.4%	25; 48.1%	
Nationality				
Saudi	44; 84.6%	262; 75.1%	1094; 73.0%	0.000
Non-Saudi	8; 15.4%	87; 24.9%	404; 27.0%	
Hospitals Name				
KAOH	1; 1.9%	21; 6.0%	991; 66.2%	0.000
KFH	45; 86.5%	256; 73.4%	507; 33.8%	
KAH	6; 11.5%	72; 20.6%	0	

Group 1: Patients have a TW Phenomenon , Group 2:Patients have a combination may use NSAID from OTC or different providers lead to TW and Group 3:patients with no drugs combination P-Value <0.05 considered significant.

Regarding gender, no notable differences between studied groups for patients' records TW phenomena (group 1); 51.9% were male and 48.1% were female; also in group 2 male was 55.0% and 45.0% female. Additionally in group 3 the male was 67.6% and 32.4% female (p-value=0.143) **Table 1**.

Most patients in all groups were from Saudi Arabia. Exactly, 84.6% for group 1, 75.1% for group 2 and 73.0% for group 3. While on the other hand, the non-Saudis were 15.4%, 24.9%, and 27.0% across the respective groups (p-value <0.001).

The data were further segmented based on the hospitals where the patients were treated. KAOH has the highest representation, 1.9% of patients experiencing the TW Phenomena (group 1), 6.0% of group 2 those with NSAID use that might be leading to TW, and accounting for 66.2% of patients without drug combinations (group3). KFH, on the other hand, represents 33.8%, 73.4%, and 86.5% across the respective groups. Notably, KAH 11.5% of group 1 patients experiencing the TW Phenomena 20.6% of patients with NSAID use leading to TW (group 2) and has no representation in group 3; the group without drug combinations but represents (p-value<0.001).

**Figure 2:**
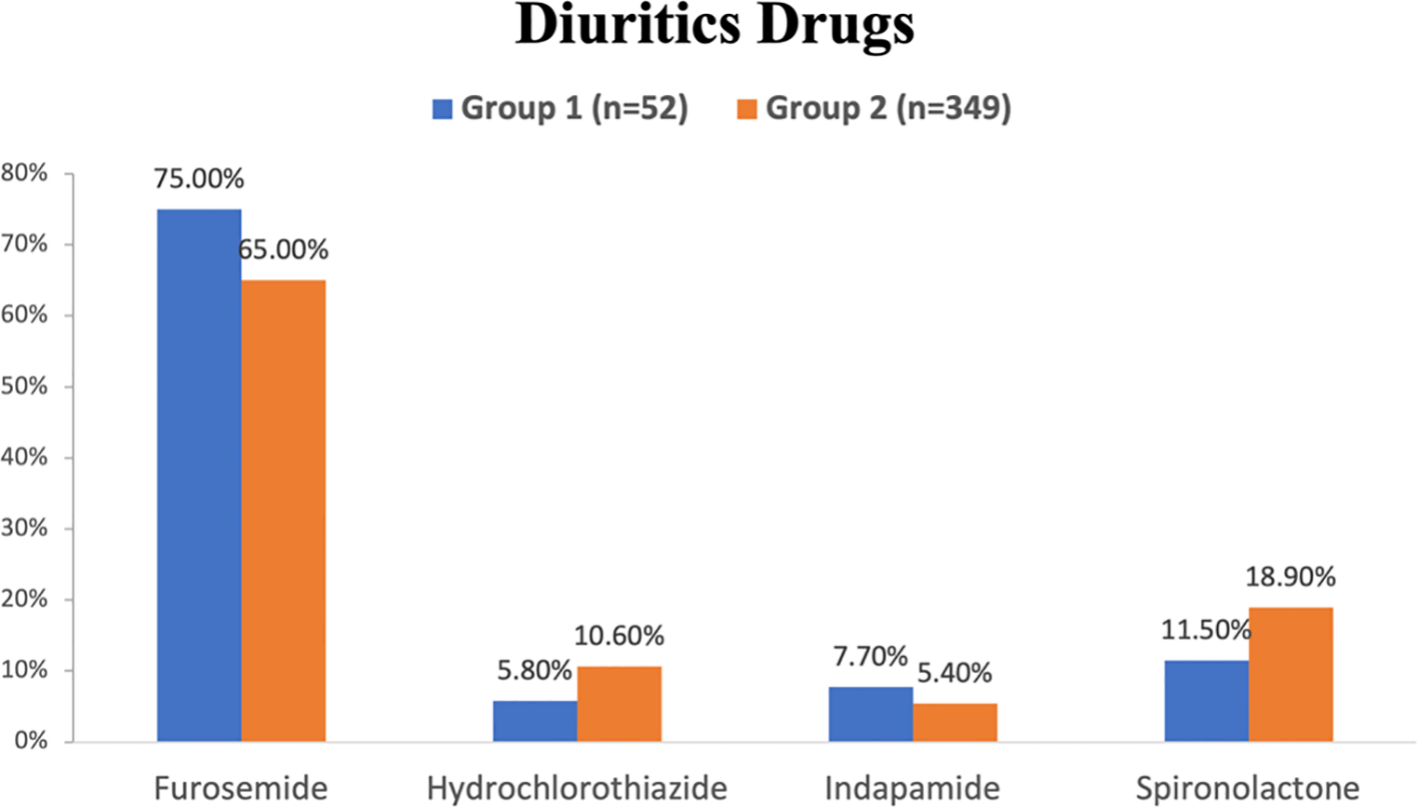
Diuretics medications that were prescribed to the patients of group 1 and 2 in the hospitals studied.

**Figure 3:**
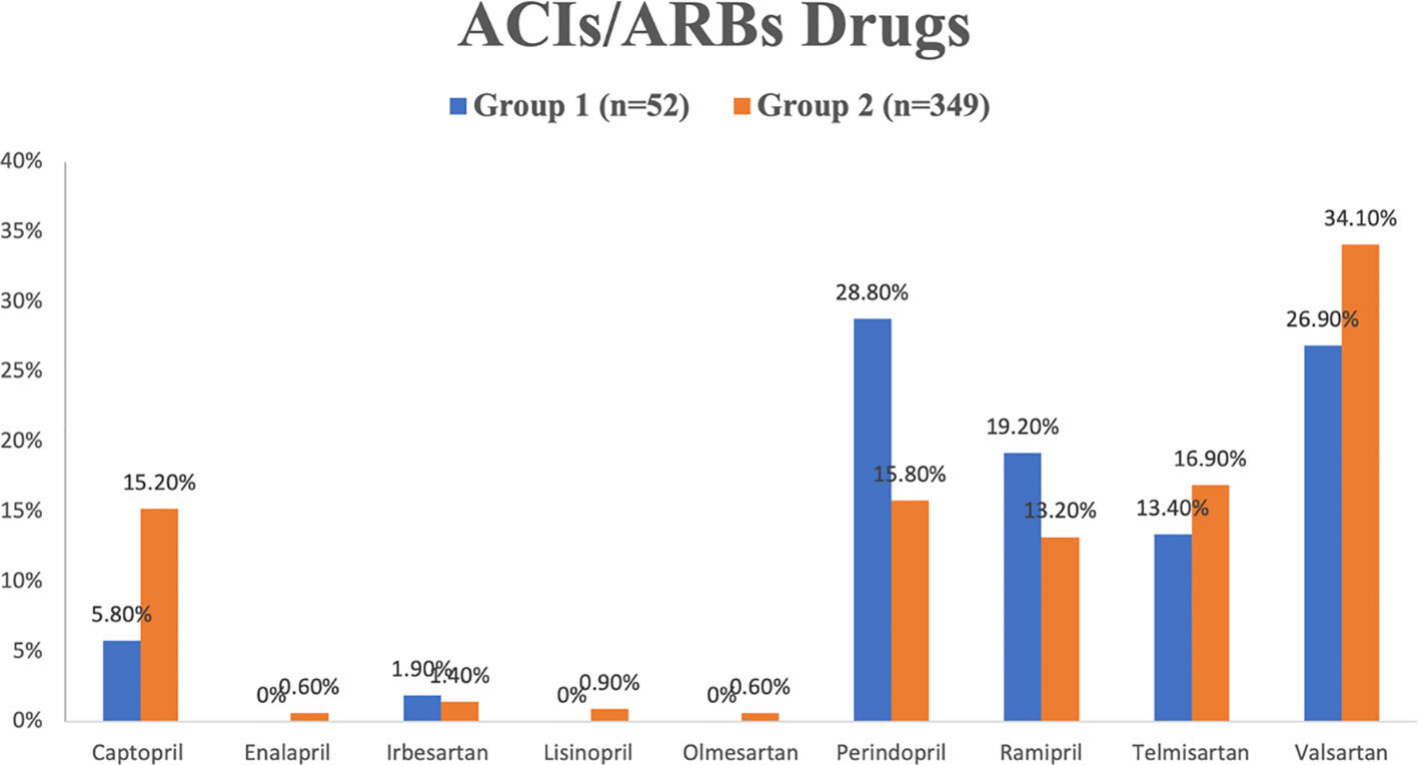
ACIs/ARBs medications that were prescribed to the patients of group 1 and 2 in the hospitals studied.

**Figure 4:**
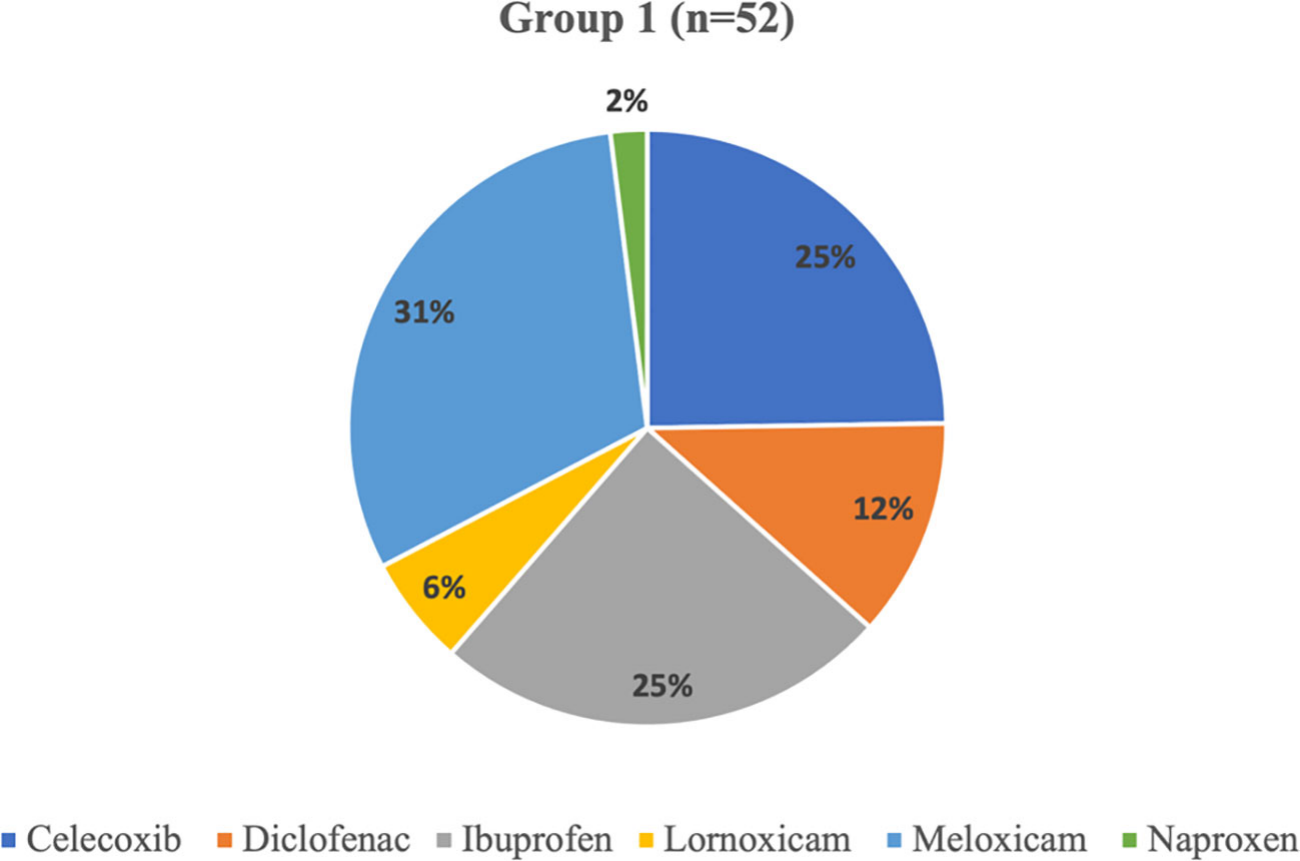
NSAIDs medications that prescribed to the patients who have the TW phenomena.

The data of different drugs that were prescribed to the patients and collected from patients’ records; which explained as the following; diuretics medications family in **Figure 2**, ACI/ARB medications family in **Figure 3** and NSAIDs in **Figure 4**.

The percentages denote the relative distribution of specific diuretics, including furosemide, hydrochlorothiazide, indapamide, and spironolactone, within all groups. furosemide exhibits the highest prevalence of diuretics that prescribed to both groups; group1 (75.0%) and group 2 (65.0%), while hydrochlorothiazide and indapamide have the least percentage of diuretics that prescribed for group 1 and group 2 respectively (**Figure 2**).

The data indicates that perindopril and valsartan were the most frequently prescribed drugs within the ACEI and ARB drug families. Specifically, perindopril accounted for 28.8% of prescriptions in group 1, while valsartan constituted 34.1% of prescriptions in group 2. Conversely, enalapril, olmesartan, and lisinopril had notably lower utilization rates, each below 1.0% in group 2, and none were utilized in group 1 (refer to **Figure 3**).

In the context of NSAIDs, in patients experiencing the TW interaction (group 1), Meloxicam emerged as the most commonly prescribed analgesic, representing 31% of utilization. In contrast, Naproxen had the lowest usage at 2%. Furthermore, Ibuprofen and celecoxib together accounted for 25% of NSAIDs prescriptions within the same group (**Figure 4**). **Table 2** depicts healthcare professionals' awareness of the TW phenomena.

**Table 2:** Awareness of Healthcare Professional Regarding TW Phenomenon.

Variables	Healthcare Providers they didn't heard about Triple Whammy phenomenan; %	Healthcare Providers they heard about Triple Whammy phenomenan;%	P-Value
Frequency	42; 75%	14; 25%	---
Nationality			
Saudi	4; 9.5%	1; 7.1%	0.79
Non-Saudi	38; 90.5%	13; 92.0%	
Gender			
Male	16; 38.1%	8; 57.1%	0.24
Female	26; 61.9%	6; 42.9%	
Years of experience			
less than 5 years	12; 28.6%	3; 21.4%	0.80
5-10 years	17; 40.5%	7; 50.0%	
more than 10 years	13; 31.0%	4; 28.6%	
Job Title			
Consultant Physician or Dentist	6; 14.3%	0	0.53
General Medicine	2; 4.8%	2; 14.3%	
Specialist Physician	6; 14.3%	1; 7.1%	
Medical Intern	3; 7.1%	0	
Pharmacist	12; 28.6%	7; 50.0%	
Pharmacist Specialist	2; 4.8%	0	
Dentist	7; 16.7%	2; 14.3%	

**Figure 5:**
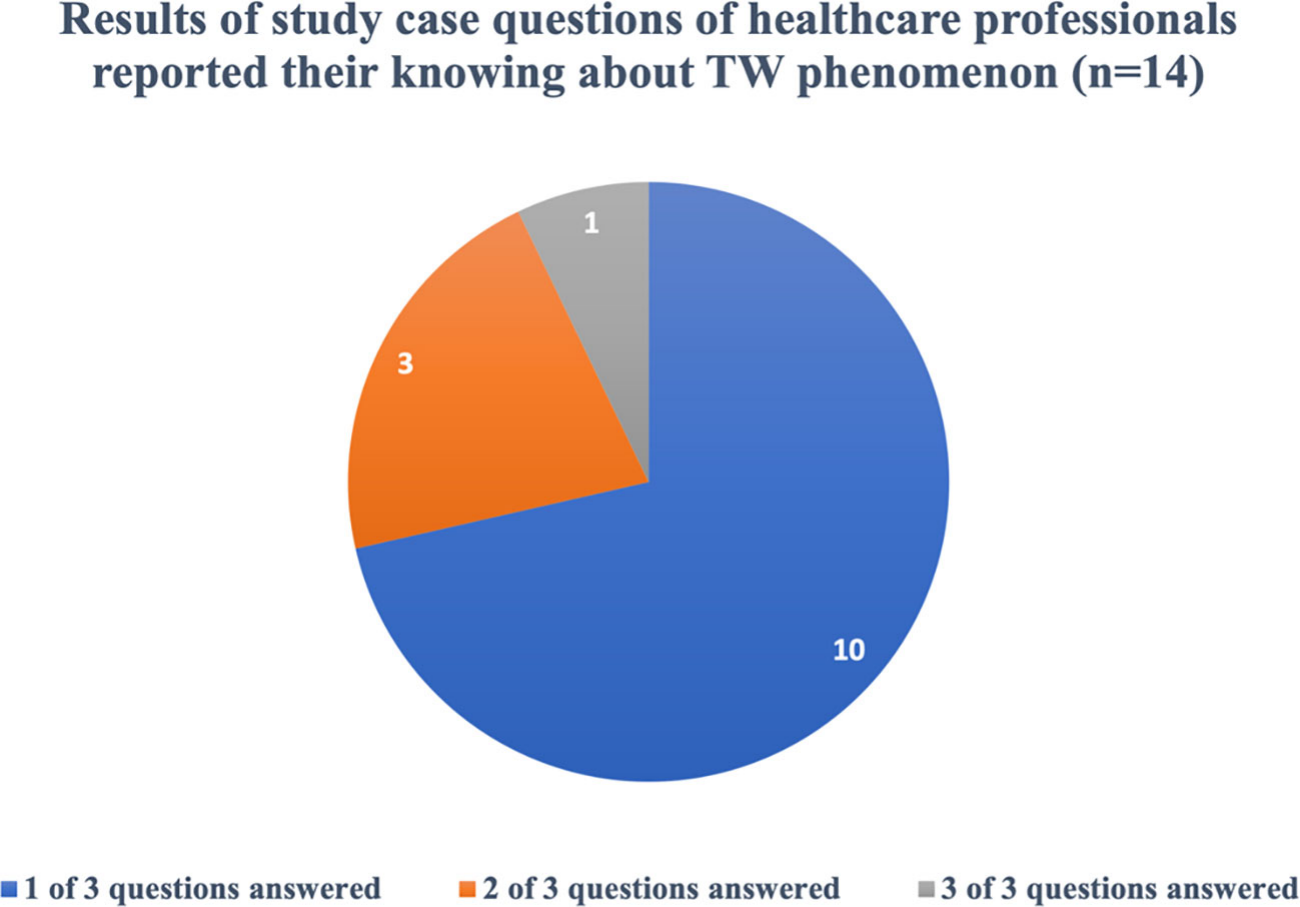
Results of study case questions that answered correctly by the healthcare professionals who reported their knowing about TW phenomenon. The analysis of nationality shows that Non-Saudi: 38 patients (90.5%) are in the unaware group. (38 out of 42), Non-Saudi: 13 (92.0%) are in the aware group. (13 out of 14).

Out of the 56 healthcare professionals who signed an informed consent and participated in the electronic survey, a significant majority, constituting 75% (N=42), were found to be unaware of the TW phenomena. On the other hand, 25% (N=14) indicated that they were familiar with TW phenomena. However, among those who claimed awareness, the majority (N=13) answered the case study questions related to TW phenomena incorrectly. Remarkably, only one healthcare professional answered all the questions correctly, as illustrated in **Figure 5**.

The statistical comparison indicates no significant difference between the two groups, with a P-value of 0.79. Furthermore, there were no significant differences observed based on the gender of healthcare professionals. Among females, 61.9% reported no awareness of TW phenomena, and among males, 38.1% reported no awareness. The lack of awareness was not statistically different between the two gender groups (P-Value=0.24).

The analysis indicates that there is no clear correlation between years of experience and awareness levels of the TW phenomena among healthcare professionals. However, specific patterns emerge when considering job titles and hospital affiliations.

Among different job titles, pharmacists were identified as the highest group lacking awareness of the TW interaction, with 28.6% reporting no knowledge. Dentists followed with 16.7%, while the remaining healthcare providers constituted the lowest group with 14.3% being unaware of TW. These findings suggest variations in awareness levels based on job titles, with pharmacists and dentists showing higher percentages of lack of awareness compared to other healthcare providers.

## Discussion

This study, which was conducted in small-scale hospitals in Jeddah (2017–2022), assessed the incidence of TW and the awareness of healthcare professionals of this condition, revealing a 2.7% TW rate and significant knowledge gaps. TW poses a serious AKI risk; however, the findings of this study highlight its underrecognition in this setting. We have revised this section to establish stronger connections between our findings and healthcare policy, education, and clinical interventions while further investigating factors that contribute to the awareness gaps among healthcare professionals.

### Linking results to broader implications

The 2.7% TW incidence and the 75% unawareness among healthcare professionals underscore the urgent need to inform clinical decision-making, healthcare policy adjustments, and educational initiatives. Our findings suggest that structured continuing medical education (CME) programs targeting recognition of TW could significantly reduce the risk of AKI, particularly for pharmacists (28.6% aware) and dentists (16.7% aware). Pharmacist-led interventions, such as medication reviews, could prevent high-risk drug interactions, even in small-scale settings such as ours. Institutional policies mandating TW risk assessments before prescribing NSAIDs, particularly for hypertensive patients on ACEIs/ARBs and diuretics, could mitigate the 2.7% incidence observed. Furthermore, we identify clinical inertia as a significant challenge in addressing the Triple Whammy phenomenon, first described by Merlin C. Thomas in the *Medical Journal of Australia* in 2000 (2). There is an opportunity for medical curricula and healthcare institutions in Saudi Arabia to enhance their training programs by incorporating this critical drug-drug interaction. This delay in knowledge dissemination has contributed to persistent awareness gaps, even among experienced providers, as shown by the lack of correlation with years of experience in our survey (*p* = 0.80).

### Factors contributing to awareness gaps

Training differences play a major role in the significant awareness deficiency observed (75% unawareness), particularly given the diverse healthcare workforce in Saudi Arabia, where over 50% of professionals are expatriates trained in various medical systems (Ministry of Health, Saudi Arabia) (14). This variability in educational backgrounds likely contributed to the inconsistent knowledge of TW risks. Standardized continuous professional development (CPD) programs could mitigate these disparities and enhance uniformity in TW awareness. Furthermore, clinical inertia—the delay in translating updated medical knowledge into clinical practice—perpetuates this gap. Many frontline healthcare workers may not have received recent updates on TW through continuing education, further exacerbating the knowledge deficit observed in this study.

Beyond training gaps, other contributing factors include the following:

- *Widespread availability of OTC NSAIDs*: OTC access to NSAIDs (67% painkiller use) (9) may have desensitized healthcare professionals to the risk of TW, leading to a less cautious prescribing behavior.
- *Institutional variations in protocols*: Differences in the institutional protocols across hospitals align with the varied TW incidence rates observed (e.g., 86.5% of group 1 at KFH), potentially contributing to the inconsistent risk assessment and management practices.
- *Nature of dentists’ scope of practice*: It is possible that the nature of the scope of practice in dentistry, which is generally confined to oral health, limits dentists’ exposure to systemic medications with higher side effects and complex interactions, such as those involved in TW (e.g., ACEIs/ARBs, diuretics, and NSAIDs leading to AKI). Dentists typically prescribe medications such as antibiotics, painkillers (including NSAIDs), anti-inflammatories, and antifungals, which are less systemic and require less knowledge of broader pharmacological risks compared with physicians who manage chronic conditions. This general focus, as reflected in our survey that showed only 16.7% TW awareness among dentists in our small-scale hospital, could have increased the risk of overlooking systemic drug–drug interactions, particularly in hypertensive patients on antihypertensive therapy, and hindered the integration of dental care with broader medical management to recognize TW risks (10).

While this study, which was conducted in small-scale hospitals, provides a foundation for identifying this awareness gap, we acknowledge the lack of direct 2024–2025 studies on TW awareness among Saudi dentists. Recent reports suggest that only 18%–22% of dentists globally recognize systemic drug interactions such as TW, which is attributed to their oral health focus and limited training on medications for chronic diseases (10). We recommend qualitative analyses (e.g., exploring the training curricula) and institutional surveys to better understand how policies, education, and practice environments influence TW awareness. These additions will strengthen the interpretation of our findings and offer a clear direction for addressing this critical issue.

### Comparative analysis

To position our findings within a global context, we incorporated comparative data from international studies benchmarking TW incidence and awareness levels. Research from North America and Europe indicates that TW-related AKI cases are more frequently identified and prevented in healthcare systems employing routine medication reviews and pharmacist oversight of NSAID prescriptions. For instance, regions with restricted availability of NSAIDs and structured involvement of pharmacists report lower TW-related hospitalizations (0.5%–1% incidence) compared with our 2.7% rate, which is likely due to the widespread OTC access of NSAIDs in Saudi Arabia. A recent cohort study on hypertensive patients with obstructive sleep apnea in China determined a J-shaped relationship between the weight-adjusted waist index (WWI) and CVD risk, suggesting that obesity metrics such as WWI may further complicate the cardiovascular outcomes in hypertension, potentially increasing the risk for patients on multiple medications such as those at risk for TW. In contrast, areas with unrestricted availability of NSAIDs and limited roles for pharmacists, similar to Saudi Arabia, report higher TW-related AKI rates. These comparisons highlight the need for regulatory adjustments in Saudi Arabia, particularly with regard to the dispensing policies for NSAIDs and interdisciplinary collaborations between physicians and pharmacists to align with global best practices.

### TW incidence and drivers

The 2.7% TW incidence (52/1,899) is lower than the up to 30% reported in critically ill patients internationally, which is likely due to our exclusion of CKD/renal failure cases and the small-scale hospital focus. However, it exceeds typical rates (e.g., 0.5%–1% in 11) for general populations, possibly due to OTC access of NSAIDs. Recent studies on hypertension management, such as those in non-high-risk populations, have highlighted that even well-controlled blood pressure can contribute to cardiovascular events if not monitored carefully, emphasizing the need for vigilance in hypertensive patients on multiple medications such as those at risk of TW. A recent cohort study on hypertensive patients with obstructive sleep apnea in China also found a J-shaped relationship between WWI and CVD risk, suggesting that obesity metrics such as WWI may exacerbate cardiovascular risks in hypertension, potentially increasing vulnerability to complications such as TW-related AKI; however, its direct relevance to NSAIDs or AKI is limited. The high TW prevalence at KFH (86.5% of group 1 and 73.4% of group 2) reflects its rural referrals and its older, male patient base (mean age of 65 ± 13.2 years, 55% men). The group 2 rate of 18.4% (349/1,899) likely underestimates true TW due to unrecorded OTC medication use. The small-scale hospital focus may have limited the generalizability of the results, necessitating broader studies. The severe outcomes of TW, including up to 50%–80% mortality in critically ill patients, and its significant AKI costs (representing 5% of hospital budgets and 1% of the health expenditure) underscore its public health impact in Saudi Arabia.

### Awareness gaps among healthcare professionals

The survey (*n* = 56) showed that 75% (42/56) of professionals were unaware of TW, with pharmacists (28.6%) and dentists (16.7%) being the most unaware, supporting training gaps. However, among the 14 aware respondents, 71.4% demonstrated full scenario-based knowledge (**Figure 5**), suggesting a knowledgeable subset. This discrepancy may have reflected a methodological overlap (e.g., *n* = 14 as a pilot or a specialist subgroup) or an underrepresentation of broader awareness. Non-Saudis (13 (92.0%) are in the aware group (13 out of 14) and 38 (90.5%) are in the unaware group (38 out of 42)) dominate, reflecting the more than 50% expatriate workforce (MOH Open Data). Having no experience/job title differences (*p* > 0.05) indicate systemic training issues, exacerbated by clinical inertia and curriculum gaps.

### Policy and education interventions

Given our findings of 2.7% TW incidence and 75% unawareness, these results should inform clinical decision-making, healthcare policy adjustments, and educational initiatives. We propose the following specific action points:

- *Mandating TW risk assessments*: Require TW risk assessments in routine clinical practice before prescribing NSAIDs, particularly for hypertensive patients on ACEIs/ARBs and diuretics, in order to prevent AKI in small-scale and larger settings.
- *Enhancing continuing education programs*: Develop and expand CME programs for healthcare providers in order to improve TW recognition and promote safe prescribing, targeting pharmacists and dentists who had the lowest awareness (28.6% and 16.7%, respectively) and implementing standardized CPD to address expatriate training disparities. For dentists, incorporate interactive pharmacology sessions and webinars, such as the 2025 Clinical Pharmacology in Dentistry Update (12), to address their limited exposure to systemic medications and enhance their understanding of TW risks, including drug interactions and chronic disease management.
- *Strengthening pharmacist involvement*: Enhance the involvement of clinical pharmacists in medication reviews in order to prevent high-risk drug interactions, leveraging their role in the mitigation of TW risks even in resource-limited hospitals.
- *Encouraging policy changes*: Advocate for policy changes, such as the re-evaluation of NSAID prescription regulations and the provision of a limit to OTC availability of high-risk NSAIDs (e.g., ibuprofen 400 mg, naproxen, and diclofenac) in hypertensive patients, aligning with Saudi Food and Drug Authority (SFDA) considerations.
- *Alternative pain management strategies*: Promote diverse approaches to pain management in order to reduce NSAID reliance and TW risk, including:

- Non-pharmacological pain relief: Encourage techniques such as physical therapy, acupuncture, heat/cold therapy, and mindfulness-based stress reduction for musculoskeletal pain, particularly in hypertensive patients.
- Addressing neuropathic pain with alternative pharmacological classes: Recommend serotonin–norepinephrine reuptake inhibitors (SNRIs), selective serotonin reuptake inhibitors (SSRIs), or anticonvulsants (e.g., gabapentin or pregabalin) for neuropathic pain, offering safer alternatives to NSAIDs for patients on ACEIs/ARBs and diuretics.

These actions build on global evidence and address Saudi-specific challenges such as OTC access, training disparities, and institutional variations while minimizing AKI risk by reducing exposure to NSAIDs.

### Recommendations for future research

To further enhance the impact of the study, we have expanded our recommendations for future research:

- *Prospective interventional studies*: Evaluate the effectiveness of educational programs, such as structured TW training for physicians and pharmacists, on reducing TW incidence and improving awareness of this condition.
- *Longitudinal studies*: Track changes in TW incidence following policy adjustments, such as NSAID prescribing restrictions or pharmacist-led risk assessment initiatives, to assess their impact on AKI rates.
- *Nationwide surveys*: Conduct a nationwide survey assessing TW awareness across different healthcare sectors, allowing for a comprehensive understanding of knowledge gaps and training needs.
- *Qualitative research*: Explore how institutional protocols, cultural prescribing habits, and training curricula influence TW risk and awareness in diverse healthcare settings, including primary health care (PHC) and rural hospitals•.
- *Hospital-level anal*yses: Specifically examine hospital-level differences, incorporating factors such as institutional policies, prescribing habits, and NSAID accessibility, in order to better understand their role in TW incidence. This would enable more tailored preventive strategies, expanding the implications of the study beyond incidence documentation.
- *Pain management studies*: Investigate the effectiveness and feasibility of non-pharmacological pain relief and alternative pharmacological classes (e.g., SNRIs, SSRIs, and anticonvulsants) in reducing NSAID use and TW incidence among hypertensive patients in Saudi Arabia, particularly in small-scale and rural settings.

The Triple Whammy phenomenon is a well-documented DDI with severe clinical implications, as it can precipitate AKI, particularly in vulnerable populations such as the elderly or those with pre-existing renal impairment. The low awareness observed in this study underscores a critical gap in healthcare providers’ knowledge of DDIs, which could contribute to preventable medical errors and adverse patient outcomes. This is particularly concerning in the context of Saudi Arabia, where the healthcare system is undergoing transformative reforms under Saudi Vision 2030, which prioritizes enhancing healthcare quality, reducing medical errors, and improving patient safety.

To address this knowledge deficit, it is imperative to integrate comprehensive education on DDIs, including the Triple Whammy phenomenon, into the curricula of the Saudi Board Certificate and training programs overseen by the Saudi Commission for Health Specialties (SCFHS). The SCFHS, as the regulatory body responsible for accrediting and training healthcare professionals in Saudi Arabia, is uniquely positioned to mandate enhanced DDI education. By incorporating modules on the Triple Whammy and other high-risk DDIs into residency and continuing professional development programs, the SCFHS can ensure that all healthcare providers—both Saudi and Non-Saudi—possessing the requisite knowledge to mitigate these risks. This would not only elevate the standard of care but also align with the objectives of Saudi Vision 2030 by fostering a healthcare workforce capable of delivering safer and more effective patient care.

Moreover, updating the Saudi Board curriculum to emphasize DDIs would serve as a proactive measure to attract and prepare healthcare providers seeking to practice in the Kingdom. A robust focus on DDI education would signal to prospective professionals that Saudi Arabia prioritizes cutting-edge, evidence-based training, thereby enhancing the Kingdom’s reputation as a hub for advanced healthcare delivery. Such an initiative could also reduce the incidence of medication-related adverse events, thereby improving health outcomes and supporting the Vision 2030 goal of building a sustainable, high-quality healthcare system.

Given the small-scale focus, these studies are critical to effectively measure the incidence of TW, especially considering its potential 30% prevalence in critically ill patients and its severe outcomes.

### Limitations

Conducted in small-scale hospitals, this study excluded rural PHC centers and other regions, limiting its generalizability. The 5-year scope may have missed longer trends, and the small survey samples (*n* = 56, *n* = 14 scenarios) may not have reflected broader awareness. The 14 scenario samples likely represent a pilot or subset, not the full workforce. No renal follow-up data precluded AKI severity analysis. In addition, the lack of recent 2024–2025 studies on TW awareness, particularly among Saudi dentists, limited direct evidence of their pharmacological training gaps.

## Conclusion

This study, which was conducted in small-scale hospitals in Saudi Arabia, highlights the 2.7% incidence of TW among hypertensive patients, driven by OTC NSAID availability and awareness gaps (75% unaware). The rural referrals and the older male patients at KFH amplify the risk. The limited sample from the small-scale hospitals (*n* = 56, *n* = 14 scenarios) underscores the need for broader research. Enhancing education (especially for pharmacists and dentists), promoting pharmacist roles, and regulating NSAIDs (e.g., reclassifying ibuprofen 400 mg, naproxen, and diclofenac as prescription-only) can reduce TW-related AKI, given its potential for up to 30% incidence and 50%–80% mortality in critically ill patients, costing 5% of the hospital budget and 1% of the health expenditure (13). Mandating TW risk assessments, strengthening continuing education, limiting OTC availability of NSAIDs, and adopting alternative pain management strategies (e.g., non-pharmacological relief, SNRIs, SSRIs, and anticonvulsants) will inform clinical decisions, policy, and education. Future national studies are essential to accurately measure the incidence of TW and improve the safety outcomes of patients.

## Data Availability

The raw data supporting the conclusions of this article will be made available by the authors without undue reservation.
